# Research on an Adaptive Coupling Technique for Spatially Scattered Light

**DOI:** 10.3390/s26061946

**Published:** 2026-03-19

**Authors:** Xin Liu, Shiyang Shen, Lei Zhu, Lisong Deng, Xiangyu Wang, Mingfeng He, Fei Xiao

**Affiliations:** 1 School of Electronic Science and Engineering, Chongqing University of Posts and Telecommunications, Chongqing 400065, China; liuxin1@cigit.ac.cn; 2 Research Center for Phased Array Optics, Chongqing Institute of Green and Intelligent Technology, Chinese Academy of Sciences, Chongqing 400714, China; gbs114@mail.ustc.edu.cn (S.S.); zhulei@cigit.ac.cn (L.Z.); denglisong149@163.com (L.D.); wangxiangyu@cigit.ac.cn (X.W.); hemingfeng@cigit.ac.cn (M.H.)

**Keywords:** underwater LiDAR, spatially scattered light, fiber coupling, SPGD algorithm

## Abstract

Focusing on the problems of difficult alignment and low efficiency when coupling the spatially scattered light from 532 nm underwater LiDAR to a single-mode fiber, this paper presents an analysis and simulation of the coupling principle of spatially scattered light and its influencing factors based on the extended light source imaging model, and designs and develops a spatially scattered light adaptive coupling system. The system adopts a three-lens set to receive spatially scattered light, combines a fast steering mirror and displacement stage to adjust the beam position dynamically, and realizes the automatic and efficient coupling of spatially scattered light through a joint control strategy combining rough alignment and precise alignment (using the improved simulated annealing SPGD algorithm). The experimental results show that the best coupling efficiency reaches 88.18% of the theoretical value after program adjustment. This represents an approximate 88% improvement over the best coupling efficiency obtained after manual adjustment, whilst the algorithm effectively circumvents the issue of local optima. This study provides a feasible adaptive solution for underwater LiDAR and similar applications involving scattered light coupling.

## 1. Introduction

Compared with sonar, underwater lidar offers a high spatial resolution and excellent directivity, with broad application prospects in marine resource exploration, underwater target ranging, and velocity measurement [1]. Due to the optical properties of water, blue–green light (450–580 nm) experiences relatively low absorption and scattering. The 532 nm wavelength exhibits low absorption and scattering coefficients, so it is often chosen as the lidar operating wavelength to minimize the influence of water on laser transmission [2,3,4,5].

In 532 nm underwater lidar systems, efficiently coupling spatially scattered light carrying target information for reception is crucial to the overall system performance [6,7]. Single-mode fiber is an ideal medium for high-sensitivity coherent reception, low-noise optical amplification and high-performance optical filtering due to its superior performance and good compatibility with most optical devices. However, the coupling process of spatially scattered light into single-mode fibers faces the challenges of difficult alignment, a low coupling efficiency, and high device accuracy requirements due to the small diameter of the mode field for single-mode fibers, the uncertainty of the distance and angle of the underwater target, and the turbulence of the water body.

In recent years, to improve the coupling efficiency of single-mode optical fiber, multifaceted studies have been conducted in the academic community. (1) Static fiber coupling technology: In 2005, Sherman M P et al. designed an optical wireless communication system utilizing a Ritchey–Chretien (RC) telescope equipped with an aspheric mirror. This system positions an n × n fiber array at the focal plane of the Ritchey–Chretien optical telescope, enabling point-to-multipoint communication with a single optical telescope [8]. However, the method is mainly applicable to static scenes. (2) Adaptive optics and dynamic coupling optimization algorithms: To enhance the coupling efficiency in dynamic environments, some scholars have carried out research in the field of adaptive optics and dynamic coupling optimization algorithms. In 2019, Li Bo et al. proposed an adaptive coupling method based on the coarse-precision laser chaptering technique, and the average coupling efficiency of the system was improved to 62.4% under a simulated atmospheric turbulence environment [9]. In the same year, Carriz et al. proposed an adaptive sequential optimization method for free-space optical communication receivers. This approach does not rely on wavefront sensors but instead achieves an effective compensation for dynamic perturbations by sequentially perturbing the detector signal after the coupler and directly maximizing the optical power coupled into the fiber using an optimization algorithm [10]. In 2025, Shuqiang Li et al. proposed a three-dimensional nutation algorithm based on a BP neural network, achieving an average coupling efficiency of 80.51% after convergence [11]. (3) Coupling technique for spatially scattered light: Spatially scattered light coupling presents unique challenges distinct from collimated beam coupling. First, the angular distribution of scattered light leads to a larger focused spot size after lens imaging, reducing the fundamental coupling efficiency. Second, the partial coherence of scattered light introduces speckle patterns that fluctuate with target motion and environmental changes. Third, the wavefront of scattered light is inherently distorted due to the random scattering process, making Gaussian mode field matching assumptions less accurate. In 2016, Ka Li et al. explored the coupling of wide-range spatially scattered light into single-mode fibers. However, their work mainly focuses on the relationship between the received field of view and the coupling efficiency, and did not deeply analyze the imaging mechanism of spatially scattered light and the factors affecting the coupling efficiency [12]. (4) Handling system uncertainties and dynamic disturbances: Fiber-optic coupling control systems are inevitably subject to system uncertainties and dynamic disturbances. This is similar to the modeling error and dynamic uncertainty challenges in complex robotic systems. Several researchers have attempted to address these challenges by employing data-driven and robust control strategies. X. Shao et al. recently demonstrated that integrating advanced learning mechanisms into dynamics modeling and motion planning can effectively handle infinite-dimensional deformations and microgravity-induced disturbances [13]. Their approach combines a practical finite-time stable optimization algorithm with a GAN-LSTM network to learn state-control mappings, thereby reducing the reliance on manual replanning. In fact, spatially scattered light coupling technology is not only crucial for underwater LIDAR but also has a wide applicability in spectral imaging, optical coherence tomography, and other detection technologies. Therefore, the development of adaptive fiber-coupling technology for spatially scattered light has significant research value and application prospects.

The coupling of spatially scattered light into a single-mode fiber can be achieved in two ways: single lens coupling and combined lens coupling. Single-lens coupling is only suitable for close-distance light source coupling, and the coupling loss for long-distance light sources is high, which makes it difficult to meet the demand for an efficient coupling of spatially scattered light [12]. Therefore, combined lens coupling has become a more common choice, and the coupling efficiency can be further improved by combining ball lenses, column lenses, and other conventional solid lenses (liquid lenses are less commonly used in practice due to gravity, which easily introduces obvious aberrations [14]).

The aim of this paper is to efficiently couple 532 nm spatially scattered light into a single-mode fiber. The main contributions of this paper are as follows. First, different from the research on collimated light, we analyze the coupling principle and influencing factors of 532 nm spatially scattered light based on the extended light source imaging model and carry out the corresponding simulation to put forward the system accuracy requirements. Second, we propose a joint optimization control strategy combining rough and precise alignment to accurately and quickly find the position of optimal coupling efficiency. Third, we propose an improved simulated annealing SPGD algorithm to avoid falling into the local maximum. In addition, we design and develop an adaptive optical coupling system and complete static and dynamic experimental tests.

## 2. Principle Analysis and Simulation

**Figure 1:**
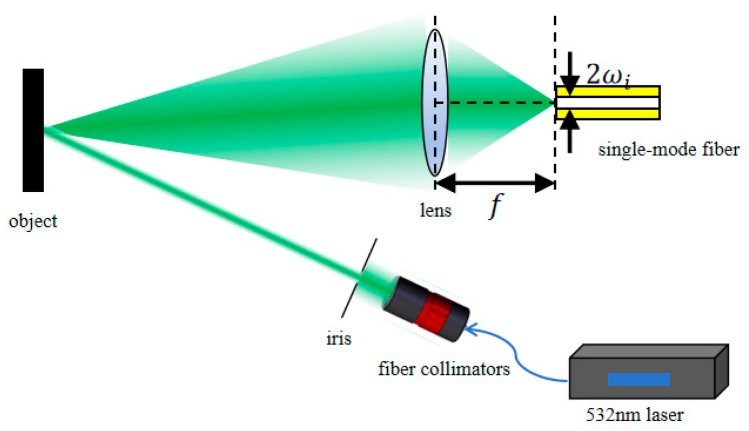
Basic structure of spatial scattering optically coupled systems.

The basic structure of the coupling system of spatially scattered light for a single-mode fiber is shown in Figure 1, which mainly consists of three parts: transmitting, scattering, and receiving. The receiving part is mainly composed of a lens and a single-mode fiber. To analyze the imaging characteristics and coupling efficiency of scattered light through the lens and their influencing factors, this paper conducts a principal analysis based on the extended light source imaging model.

### 2.1. Extended Light Source Imaging Principle Analysis

**Figure 2:**
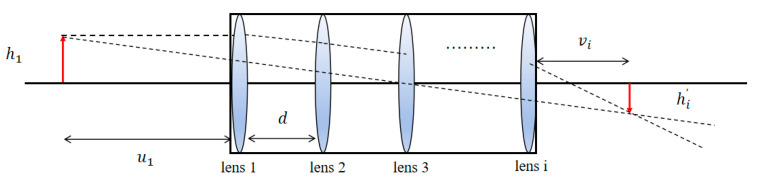
Combined lens extended light source model.

The coupling efficiency of a single-mode fiber is extremely sensitive to the spot quality and position, and, in the design of a coupling system, a single lens is usually suitable for the focusing of a close-range light source, but the coupling loss of a long-range light source is large, making it difficult to adapt to the needs of underwater applications [12,15,16]. In contrast, the combined lens can effectively reduce the coupling loss of a long-distance light source and is thus more suitable for the single-mode fiber-coupling system for underwater lidar. The extended light source imaging model based on the combined lens is shown in Figure 2.

**Figure 3:**
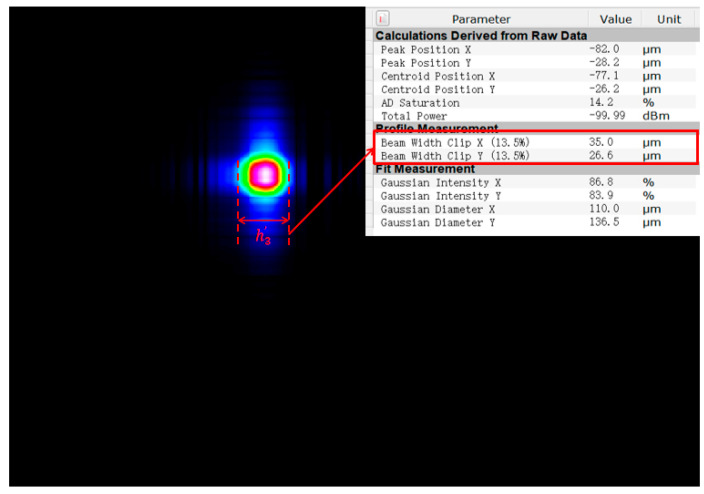
Schematic diagram of spot size after actual focusing.

Assuming that the spacing between lenses is *d*, the Gaussian imaging formula yields

(1) $$v_{i}=\frac{u_{i}f_{i}}{u_{i}-f_{i}}$$

(2) $$v_{i+1}=d-v_{i}$$

(3) $$h_{i-1}^{\prime }=h_{i}$$

(4) $$h_{i}^{\prime }=\frac{h_{i}v_{i}}{u_{i}}$$

where $v_{i}$ is the image distance imaged by the *i*th lens, $u_{i+1}$ is the object distance of the *i*+1th lens, $h_{i}$ is the object height of the *i*th lens, and $h_{i}^{\prime }$ is the image height of the *i*th lens. The spot size at the focal point of the spatially scattered light after passing through the combined lens can be calculated using Equations (1)–(4). With a fixed aperture, a lens with too short a focal length introduces significant aberrations, whereas a lens with too long a focal length increases the focused spot size. Both effects reduce the coupling efficiency and hinder the performance required for underwater LiDAR applications [17,18]. In order to avoid the impact of a single lens, the receiving system in this paper will use three identical lens combinations, a lens aperture of 25.4 mm, a single lens focal length of *f* = 150 mm, and an adjacent lens spacing of d = 10 mm, assuming that the initial object distance u_1_ = 3.8 m, the diaphragm limitation under the object height h_1_ = 2.44 mm, and the calculation of $h_{3}^{\prime }$ = 35.64 μm. That is, after the system focuses on the spot size after the actual system focusing, as shown in Figure 3, the spot size is about 35 μm, which is in line with the theoretical calculations.

### 2.2. Analysis of Spatially Scattered Light Fiber Coupling Principle and Influencing Factors

**Figure 4:**
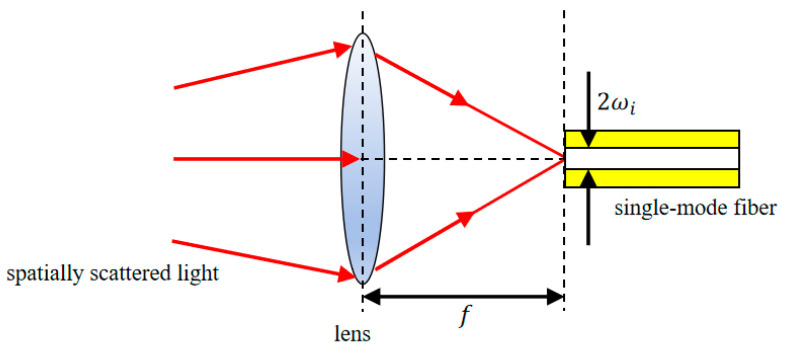
Schematic diagram of single-mode fiber coupling.

By confining transmission to the fundamental mode, the single-mode fiber effectively preserves the laser’s coherence and beam quality. Under bending or environmental perturbation, the mode distribution of the single-mode fiber is stable, and it is not easy to excite higher-order modes, which reduce the signal fluctuation and noise caused by mode coupling and help to improve the reliability of the return signal, so single-mode fibers have been widely used in the underwater LIDAR system [19]. The basic principle of fiber coupling is based on the optical field matching theory, which aims to match the Airy spot mode field incident to the end face of the fiber with the mode field of the single-mode fiber so as to achieve efficient energy coupling [20,21], and the specific structure is shown in Figure 4.

The coupling efficiency is calculated using the following equation [22]:

(5) $$\eta_{0}=\frac{{\left|\iint E_{i}\left(x,y\right)E_{f}^{*}\left(x,y\right)dxdy\right|}^{2}}{\iint {\left|E_{i}\left(x,y\right)\right|}^{2}dxdy\iint {\left|E_{f}\left(x,y\right)\right|}^{2}dxdy}$$

where $E_{i}(x,y)$ is the mode field distribution of the incident light at the fiber end face; $E_{f}(x,y)$ is the mode field distribution of the single-mode fiber. If the incident light is approximated as Gaussian light using the Gaussian approximation of the mode field distribution of the single-mode fiber, then $E_{i}(x,y)$, $E_{f}(x,y)$ can be expressed as

(6) $$E_{i}\left(x,y\right)=\exp\left(-\frac{x^{2}+y^{2}}{\omega_{i}^{2}}\right)$$

(7) $$E_{f}\left(x,y\right)=\exp\left(-\frac{x^{2}+y^{2}}{\omega_{f}^{2}}\right)$$

where $\omega_{i}$ is the mode field radius of the incident light at the fiber end face, and $\omega_{f}$ is the mode field radius of the single-mode fiber; unlike collimated light, the spot mode field radius after space-scattered light is focused by the lens needs to be calculated using Equations (1)–(4). Substituting $E_{i}(x,y)$, $E_{f}(x,y)$ into Equation (5), we obtain

(8) $$\eta_{0}=\left(\frac{2\omega_{i}\omega_{f}}{\omega_{i}^{2}+\omega_{f}^{2}}\right)$$

The primary parameters of the commonly used 532 nm single-mode fiber are listed in Table 1:

As shown in the table above, the mode field radius of the 532 nm single-mode fiber is approximately 1.65 μm, i.e., $\omega_{f}=1.65\;\mu m$. Substituting $\omega_{i}=h_{3}^{\prime }/2=17.82\;\mu m$ into Equation (8) yields $\eta_{0}=3.37\%$.

It should be emphasized that *η*_0_, as given by Equation (8), represents an idealized theoretical upper bound. This derivation relies upon several critical assumptions: (1) the light field incident upon the fiber end face exhibits a perfectly Gaussian distribution; (2) this light field is perfectly matched to the intrinsic mode field of the single-mode fiber (i.e., no wavefront aberration, no misalignment); (3) the system exhibits no aberrations whatsoever. However, in practical systems, the spatial distribution of the light field after the focusing of the lens assembly may deviate from the ideal Gaussian model due to lens aberrations, the spatial coherence of the scattered light, and speckle effects. Consequently, the $\eta_{0}$ calculated from Equation (8) is typically unattainable in practice and serves solely as a benchmark for evaluating system performance and the efficacy of alignment algorithms.

During actual coupling, a misalignment between the incident light and the fiber is unavoidable, and such deviations significantly affect the coupling efficiency of single-mode fibers. Common misalignment types include axial, radial, and angular deviations, each deviation varying in its degree of influence on the coupling efficiency [23].

**Figure 5:**
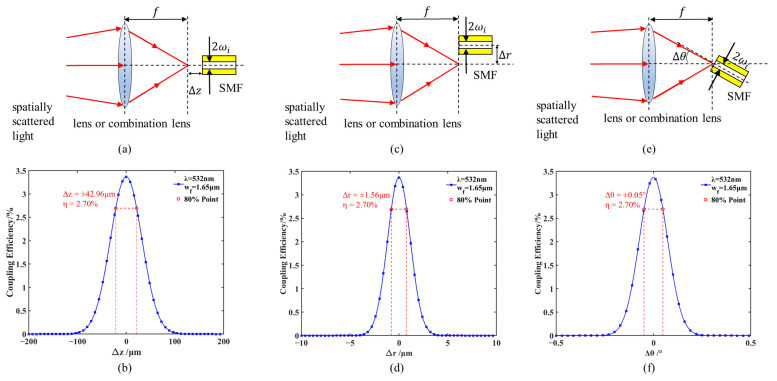
Schematic and simulation of the effect of deviations on coupling efficiency. (**a**,**b**) Axial deviation; (**c**,**d**) radial deviation; (**e**,**f**) angular deviation.

When the focal plane of the incident light does not coincide with the fiber end face, an axial offset Δz exists, known as axial deviation or defocus. A schematic of this axial offset is shown in Figure 5a. The coupling efficiency in the presence of this axial offset can be approximated as follows [23]:

(9) $$\eta_{\Delta z}=\eta_{0}\exp\left(-\frac{\lambda^{2}{\Delta z}^{2}}{8\pi^{2}\omega_{f}^{4}}\right)$$

where λ is the wavelength of the incident light.

When the incident optical axis is parallel to but not coincident with the optical axis of the fiber end face, there is a radial deviation Δ*r* between the two, which is shown schematically in Figure 5c. At this time, the coupling efficiency can be approximated as follows [24]:

(10) $$\eta_{\Delta r}=\eta_{0}\exp\left(-\frac{{\Delta r}^{2}}{\omega_{f}^{2}}\right)$$

The angular deviation occurs when there exists an angle Δ*θ* between the incident optical axis and the optical axis of the fiber end face, which is shown schematically in Figure 5e. At this time, the coupling efficiency can be approximated as follows [25]:

(11) $$\eta_{\Delta \theta }=\eta_{0}\exp\left(-\frac{\pi^{2}{\Delta \theta }^{2}\omega_{f}^{2}}{\lambda^{2}}\right)$$

Take $\lambda =532\;nm,\;\omega_{f}=1.65\;\mu m,\;\eta_{0}=3.37\%$ to simulate Equations (9)–(11); the results are shown in Figure 5b,d,f.

According to the analysis of Figure 5b,d,f, in order to realize a coupling effect of not less than 80% of the theoretical coupling efficiency, the axial deviation, radial deviation, and angular deviation need to meet Δ*z* < 42.96 μm, Δ*r* < 1.56 μm, and Δ*θ* < 0.05°, respectively; among them, the coupling efficiency is the most sensitive to the radial deviation, and thus the corresponding calibration device has the highest accuracy requirement. In order to ensure that such high precision requirements can be realized, the subsequent device selection needs to focus on its repeatability, stability and thermal drift compensation ability. If the device performance does not meet the requirements, it will seriously affect the coupling effect.

## 3. System Design and Workflow

### 3.1. System Design

**Figure 6:**
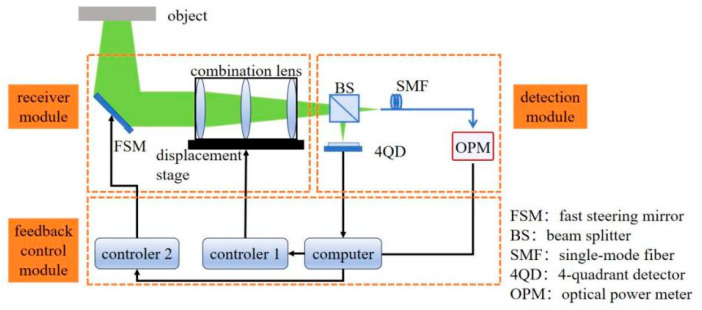
Schematic diagram of system structure.

To realize the automatic, efficient coupling of spatially scattered light, an adaptive fiber-optic coupling system is designed in this paper, as shown in Figure 6. The system consists of three main modules: receiving, detection, and feedback control.

The receiving module is responsible for receiving scattered spatial light and adjusting the beam position. It is composed of a fast steering mirror (FSM, Coremorrow, Harbin, China), a combination lens (Lbtek, Changsha, China), and a displacement stage (Xeryon, Leuven, Belgium). The detection module is used to detect the beam position and incoming fiber-optic power, including a Beam Splitter (BS, Lbtek, Changsha, China), 4-Quadrant Detector (4QD, Keyang, Suzhou, China), and Optical Power Meter (OPM, Thorlabs, Newton, NJ, USA). The feedback control module adjusts the actuators in the receiving module according to the detection information and closed-loop processing by the computer to achieve an optimal coupling efficiency, including a computer, an FSM controller (controller 2, Coremorrow, Harbin, China), and a displacement stage controller (controller 1, Xeryon, Leuven, Belgium).

According to the simulation in Figure 5, the system needs to meet the accuracy requirements of axial deviation Δz < 42.96 μm, radial deviation Δr < 1.56 μm, and angular deviation Δθ < 0.05°, which leads to the need to consider thermal drift compensation, long-term stability, and high accuracy in the selection of devices. The FSM adjusts radial and angular deviations, offering a radial accuracy <0.105 μm, an angular accuracy <0.00004°, and a positional deviation <20 nm during power cycling, and is equipped with a thermal drift compensation algorithm in controller 2; the displacement stage corrects axial deviations with an accuracy of 78 nm and a positional deviation <1 μm during power cycling, incorporates a closed-loop sensor, and is equipped with a thermal drift compensation algorithm in controller 1. The devices and their core performance parameters in each module are shown in Table 2.

### 3.2. System Workflow

The spatially scattered light carrying the target information is reflected by the FSM, focused by the lens set, and then split by the BS. The 4QD receives the reflected light for detecting the beam position; the transmitted light directly enters the fiber coupling end face and is connected to the OPM to monitor the coupling optical power; the beam offset measured by the 4QD and the optical power information obtained by the OPM are transmitted to the computer in real time, and the control commands generated by the control algorithms are processed and sent to controller 2 and the displacement stage, respectively, which dynamically adjust the beam position until the optimal coupling efficiency is obtained, and the whole workflow is completed.

**Figure 7:**
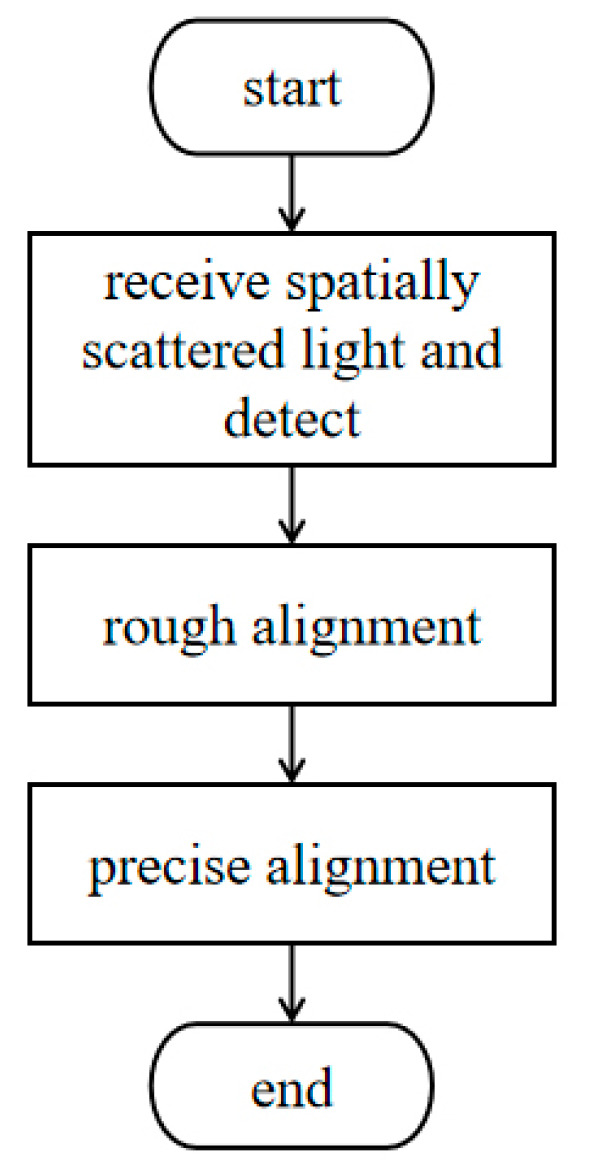
System workflow diagram.

To automatically and accurately find the optimal coupling efficiency position, this paper adopts a joint optimization control strategy combining rough and precise alignment. In the rough alignment stage, based on the beam offset calculated by the 4QD, the FSM is directly controlled to realize the preliminary alignment, which dramatically reduces the search range; in the precise alignment stage, the improved simulated annealing SPGD algorithm is used to carry out a fine search based on rough alignment to converge to the position of the optimal coupling efficiency accurately. The workflow is illustrated in Figure 7.

#### 3.2.1. Rough Alignment

If the radial deviation is too large, direct precise alignment may fail to converge to the optimal coupling position and may significantly prolong the runtime; therefore, rough alignment is performed before precise alignment, which can effectively narrow the search scope and improve the optimization speed. Given that radial deviation has the most significant effect on the coupling efficiency, the rough alignment stage is corrected only for radial deviation.

**Figure 8:**
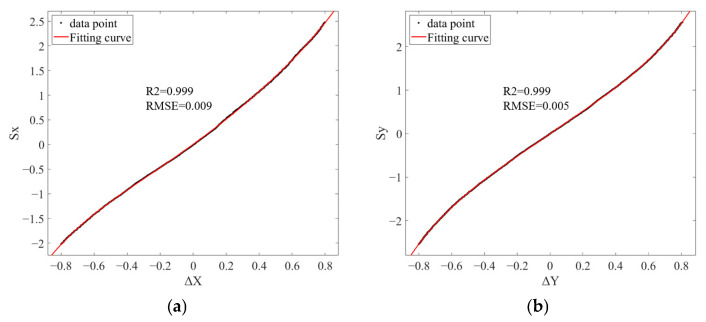
Data fitting plot. (**a**) X-axis; (**b**) Y-axis.

First, control the FSM to set the 4QD reading along the X-axis to Δ*X* = −0.8. Then, control the FSM to move along the X-axis in steps of Δ*S_x_* = 100, simultaneously recording the offset data Δ*X* measured by the 4QD. Stop when Δ*X* = +0.8; at this point, approximately 450 data points are obtained. Perform a fifth-order polynomial fit on these calibration points. The data fitting plot is shown in Figure 8a, and the fitted equation is given by Equation (12).

(12) $$S_{x}=-1050.8\Delta X^{5}-1285.4\Delta X^{4}-3650.2\Delta X^{3}-1120.7\Delta X^{2}+12030\Delta X$$

Similarly, the fifth-order polynomial fitting data plot for the Y-axis is shown in Figure 8b, with the fitted equation given by Equation (13):

(13) $$S_{y}=42.84\Delta Y^{5}-153.27\Delta Y^{4}-232.58\Delta Y^{3}-117.18\Delta Y^{2}+16121.75\Delta Y$$

**Figure 9:**
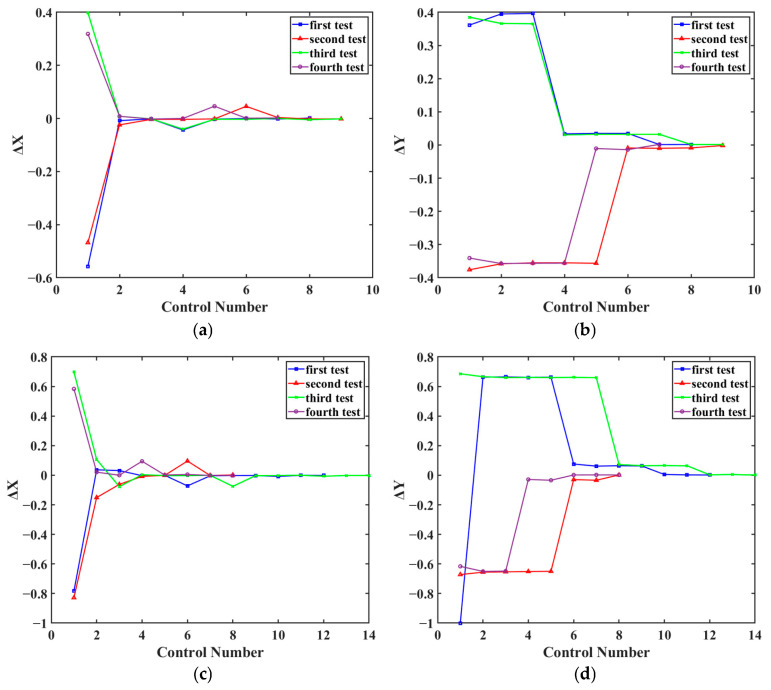
The result plots of the consistency test of the fitted equation. (**a**) x-axis offset by 0.5°; (**b**) y-axis offset by 0.5°; (**c**) x-axis offset by 1°; (**d**) y-axis offset by 1°.

As shown in Figure 8, the X-axis fitting equation achieved an R^2^ = 0.999 and an RMSE = 0.009, while the Y-axis fitting equation achieved an R^2^ = 0.999 and an RMSE = 0.005. To validate the fitted formula, the FSM was first adjusted to set both the Δ*X* and Δ*Y* outputs from the 4QD to zero. Subsequently, the collimator was deflected by 0.5° and 1°, respectively. The corresponding *S_x_* and *S_y_* values were calculated using the fitted formula and transmitted to controller 2 to drive the FSM. The verification results are shown in Figure 9. These results demonstrate that, when the collimator is deflected by 0.5° and 1°, respectively, the control parameters calculated based on the fitted formula can reduce the Δ*X* and Δ*Y* outputs of the 4QD to less than 0.1 within 10 adjustments. This confirms the validity of the fitted formula.

Lower-order polynomial fitting was also tested but resulted in higher RMSE values, 0.092 for first-order, 0.052 for second-order, 0.013 for third-order, and 0.011 for fourth-order, which did not meet the requirements. If this low-order polynomial fitting function is used, it will increase the convergence time of the rough alignment and decrease the alignment accuracy.

**Figure 10:**
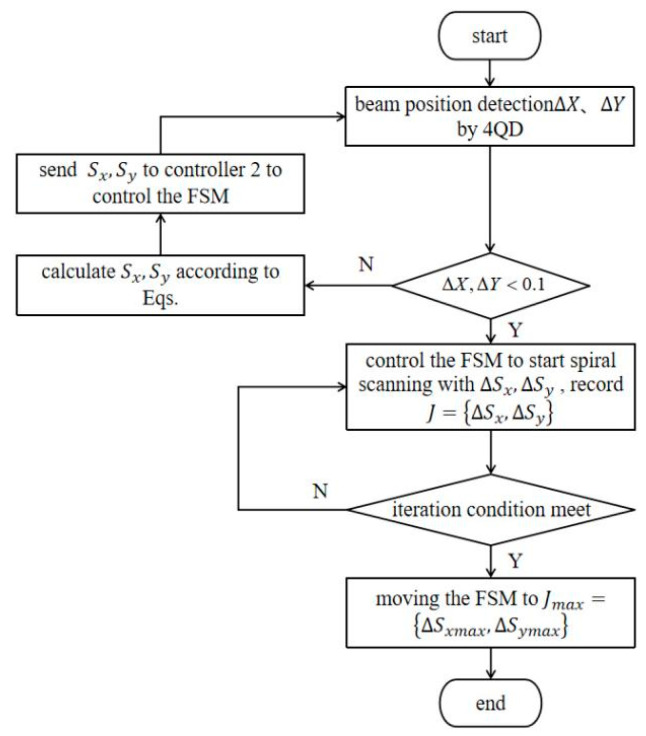
Rough alignment diagram.

In the actual control process, the rough alignment algorithm first calculates the corresponding moving steps $S_{x}$ and $S_{y}$ of the FSM based on the offsets ΔX and ΔY output from the 4QD. It sends them to controller 2 to drive the beam until the 4QD outputs of ΔX and ΔY are both less than 0.1, at which point it stops. Subsequently, a small spiral scan is performed at the position, and the coupled optical power at each point during the scan is recorded. After the scanning is finished, the position with the highest coupling efficiency is selected as the starting point for the precise alignment. The control beam is moved to that point, and the rough alignment process is complete. The specific flow of rough alignment is shown in Figure 10.

#### 3.2.2. Precise Alignment

The Stochastic Parallel Gradient Descent (SPGD) algorithm is an optimization algorithm that does not require a system model and has parallel characteristics [26,27]. The algorithm avoids the complex gradient computation process by applying stochastic parallel perturbations to the control system and observing the changes in performance metrics to estimate the gradient direction. The traditional SPGD algorithm is prone to local optima or oscillations due to the use of fixed gain coefficients and perturbation amplitudes, as well as a single gradient estimation method, which makes it difficult for the evaluation function to converge. In the scenario described in this paper, due to environmental factors, multiple local extreme points may occur near the global optimal coupling position, and it is often difficult to accurately converge to the global optimal position using the traditional SPGD algorithm.

This paper employs the optical power coupled into a single-mode fiber as the performance index J for the SPGD control algorithm. We refine the conventional SPGD algorithm to propose an improved simulated annealing SPGD algorithm. The algorithm starts from the position at the end of the rough alignment. Specifically, after completing the rough alignment, the parameter vector $S=\{s_{x},s_{y},s_{z}\}$ is the initial value S_0_ for the precise alignment algorithm. During precise alignment, the algorithm finds the optimal coupling efficiency position by coordinating the FSM parameters $s_{x}$ and $s_{y}$ and the displacement stage parameter $s_{z}$. Algorithm 1 shows the definition of the parameters in the algorithm, the setting of the initial values of the parameters and the specific procedure of the whole algorithm.
**Algorithm 1:** The Improved Simulated Annealing SPGD Algorithm Procedure$S_{k}=\{s_{x},s_{y},s_{z}\}$: initial parameter vector
$\delta S_{k}=\{\delta s_{x},\delta s_{y},\delta s_{z}\}$: random perturbation vectors
*J*: performance index (Coupled optical power)
$∆=\{{∆}_{x},{∆}_{y},{∆}_{z}\}$: initial disturbance amplitude
$G_{k}$: the gradient
γ: the gain coefficient
D: learning rate
T: acceptance probability (initial temperature)
max_iter: maximum number of iterations
tol: limit of convergence


1. Initialisation of $\delta S_{k},S_{k}$, Δ, set up the value of γ = 1, D = 0.7, T = 1, max_iter = 400, set up the saturation limits for each actuator: $|s_{xmax}|=30000,|s_{ymax}|=3000,|s_{zmax}|=10$

2. Set the initial perturbation step size: $∆=\{{∆}_{x}=1000,{∆}_{y}=1000,{∆}_{z}=0.1\};$

3. get the value of $S_{0}=\{s_{x},s_{y},s_{z}\}$

4. Measure the current evaluation function $J_{0}$:$J_{0}$= get($S_{0}$);
5. Set the iteration count: k = 0;
6. Update convergence indicator: converged = false
7. while not converged do
8.     rx = random_vector();        ry = random_vector();        rz = random_vector();Generate a random number conforming to the symmetric Bernoulli distribution, yielding either +1 or −1.9.     $\delta S_{k}=\left\{\delta s_{x},\delta s_{y},\delta s_{z}\right\}=\{rx∙{∆}_{x},ry∙{∆}_{y},rz∙{∆}_{z}\}$

10.     $S_{k}^{+}=S_{0}+\delta S_{k}$
the positive perturbation11.     $J_{k}^{+}=get(S_{k}^{+})$

12.     $S_{k}^{-}=S_{0}-\delta S_{k}$
the negative perturbation13.     $J_{k}^{-}=get(S_{k}^{-})$

14.     $\delta J_{k}=J_{k}^{+}-J_{k}^{-}$

15.     $G_{k}=\delta J_{k}*\delta S_{k}$
compute the gradient16.     $S_{k+1}=S_{0}+\gamma *G_{k}$

17.     $J_{k+1}=get(S_{k+1})$

18.     if $J_{k+1}$ > $J_{k}$ then
19.          $S_{0}=S_{k+1}$

20.          $J_{0}=J_{k+1}$

21.          γ=γ×D

22.          ${\delta S}_{k+1}={\delta S}_{k}\times D$

23.     else
24.          rnd = random(0,1)principles of simulated annealing25.          if rnd < T×D then
26.               γ=γ/D

27.               ${\delta S}_{k+1}={\delta S}_{k}/D$

28.               T = T×D

29.          end if
30.     end if
31.     k++
32.     if (abs($J_{k+1}-J_{0}$) < tol) or (k >= max_iter) thenConvergence criterion33.          converged = true
34.     end if
35. end while


**Figure 11:**
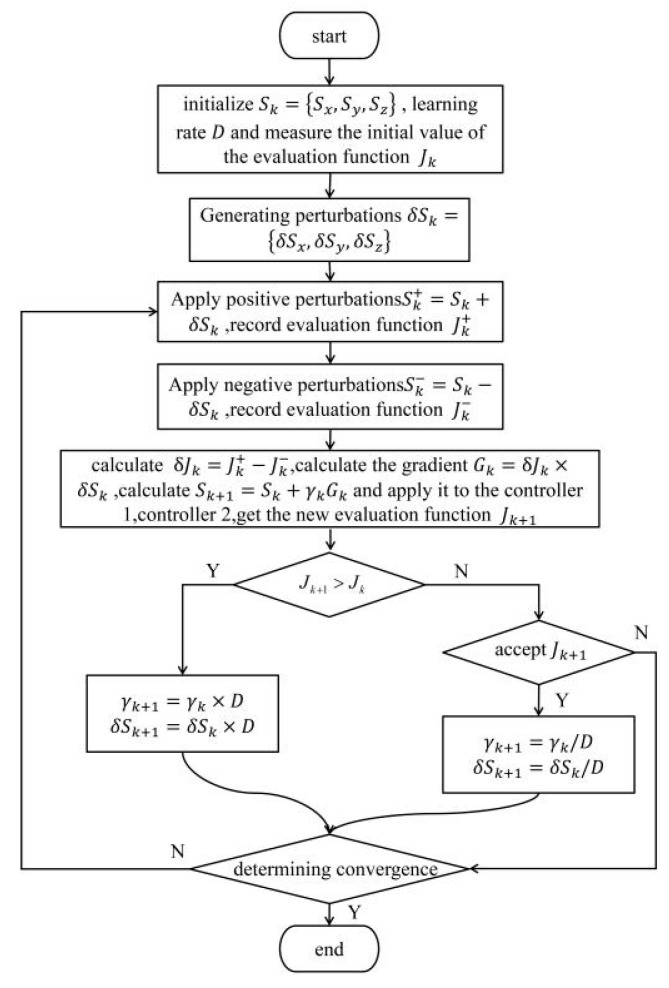
Improved simulated annealing SPGD algorithm.

This modification allows the perturbation magnitude $\delta S_{k+1}$ to decrease as the evaluation function J converges, reducing late-stage fluctuations. Simultaneously, the simulated annealing strategy helps to avoid local optima. The improved algorithm flow is illustrated in Figure 11.

**Figure 12:**
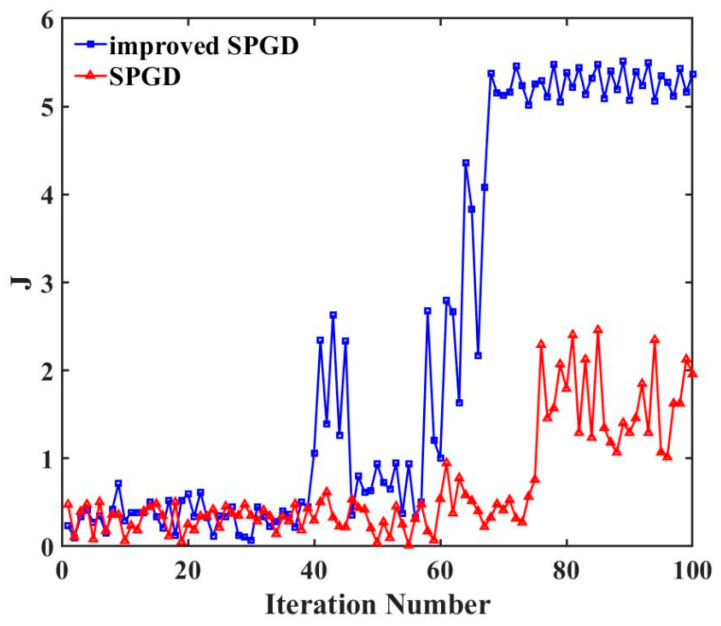
Algorithm simulation comparison chart.

In simulations, we used a multi-peak function (global max 5, local max 1.8) with added noise to compare the traditional SPGD algorithm and the improved simulated annealing SPGD algorithm, as shown in Figure 12. As can be seen in Algorithm 1, the improved simulated annealing SPGD algorithm involves a number of key parameters, including the gain coefficient γ, the initial disturbance amplitude Δ, and the learning rate D. The selection of these parameters has significant effects on the convergence speed, stability, and global optimization ability of the algorithm:

1. Gain coefficient γ and initial disturbance amplitude Δ: γ determines the update amplitude of the parameter vector *S_k_*, and Δ affects the accuracy of gradient estimation. A γ that is too large may lead to oscillations or even divergence during convergence; a value that is too small may lead to slow convergence and may fall into local minima; a Δ that is too large may lead to slow convergence; a value that is too small may lead to convergence that is too fast to find the optimal coupling position.
2. Learning rate D: In order to evaluate the impact of the D value on the performance of the algorithm, we have conducted a D value sensitivity simulation test. The results show that, when D is in the range of 0.6–0.8, the algorithms are able to converge to the vicinity of the maximum value of 5; when D < 0.6, there is an obvious oscillation in the convergence process and the best coupling position cannot be found; when D > 0.9, the convergence speed decreases significantly (>10 s). Therefore, D = 0.7 is a better choice under the current conditions.

## 4. Analysis of Experimental Results

### 4.1. Experimental Setup and Procedure

**Figure 13:**
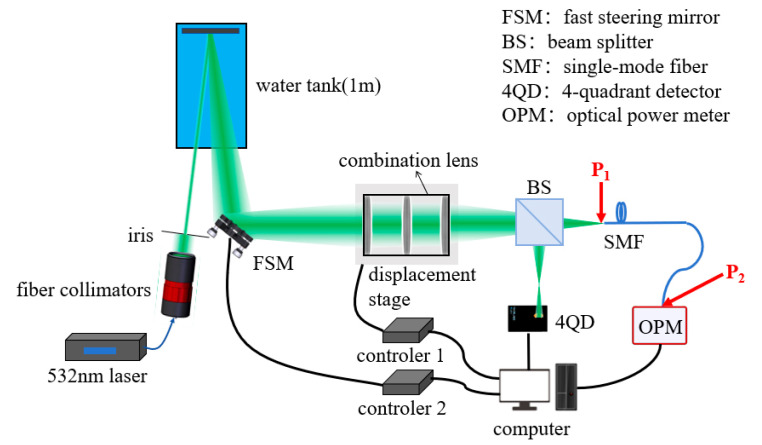
Adaptive fiber-optic coupling system.

**Figure 14:**
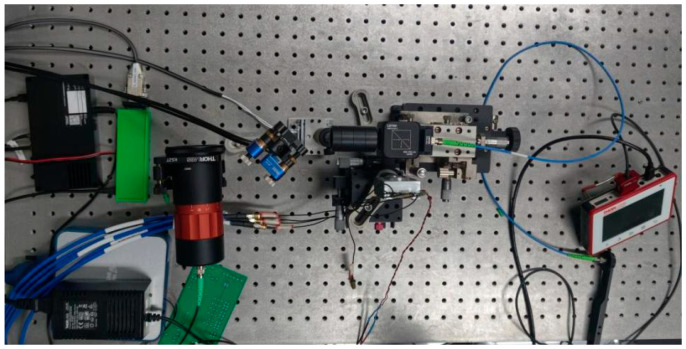
Experimental setup.

**Figure 15:**
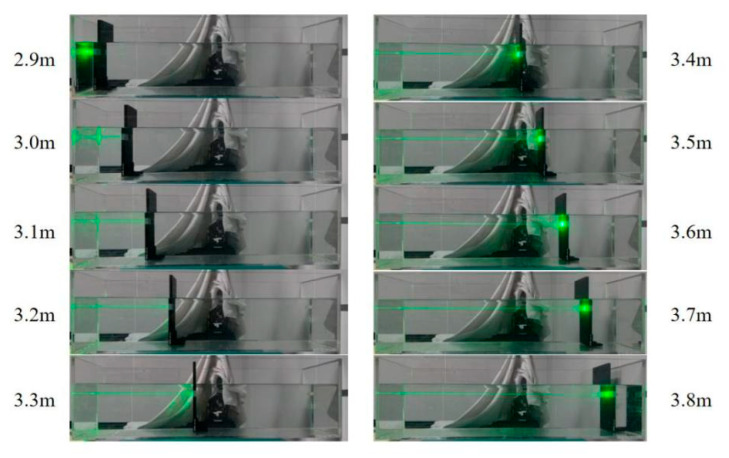
Schematic diagram of diffusely reflecting plate position.

Figure 13 illustrates the experimental setup. A 532 nm laser source with an output power of 102 mW was used in the experiment. Its output light was first introduced into a fiber collimator (C80APC-A) via a fiber, then collimated and passed through an aperture before illuminating a diffusely reflecting plate inside the water tank. The FSM reflects the scattered light and focuses it with a set of lenses mounted on a displacement stage. This beam undergoes 1:9 splitting by a BS, with the reflected portion entering the 4QD for beam position detection, while the transmitted portion is coupled into an optical fiber. The coupled optical signal is transmitted via a fiber to an OPM for measuring coupled optical power. The fiber is mounted on a five-axis precision stage for manual alignment. The actual experimental setup is shown in Figure 14. To simulate reflections from objects at varying underwater distances and angles, the diffusely reflecting plate was positioned at different locations and angles within the water tank during experiments, as illustrated in Figure 15.

Before the experiment, we calibrated the OPM and assessed its measurement uncertainty using the method provided by Thorlabs: (1) A standard 532 nm light source with an output of 5 mW was connected to the OPM. (2) The OPM wavelength was set to 532 nm. (3) The OPM reading was recorded. (4) The OPM reading was compared and calibrated against the standard 532 nm light source output. After calibration, the OPM output was 4.99 ± 0.15 mW, with a measurement uncertainty of ±3%. Subsequently, the BS underwent splitter ratio verification using the same standard 532 nm light source. Transmitted and reflected powers were directly measured using the calibrated OPM, and ten replicate tests were conducted. The average transmittance (T) was 90.1%, and the average reflectance (R) was 9.9%. The relative standard deviation of the T/R ratio was less than 1.1%.

In the experiment, the theoretical maximum coupling efficiency is denoted as *η*_0_, the optical power at the front face of the SMF is denoted as *P*_1_, and the optical power coupled into the SMF is denoted as *P*_2_. The coupling efficiency *η* is defined as $\eta =P_{2}/P_{1}$, where *P*_2_ is measured directly by the OPM connected to the back end of the SMF, and *P*_1_ is measured by the OPM directly at the front face of the SMF after precise alignment. The measurement positions of *P*_1_ and *P*_2_ are marked in Figure 13.

In the static experiment, the laser beam passed through a transparent water tank and illuminated a diffusely reflecting plate. The diffusely reflecting plate was moved in 0.1 m over the range 2.9 to 3.8 m. At each position, the initial fiber position was first recorded. Subsequently, manual adjustment using a five-axis precision displacement stage was performed to achieve the optimal coupling position; then, *P*_1_, *P*_2_ and *η* were recorded. Thereafter, the fiber was reset to its initial position. Automatic adjustment was then performed using an algorithm to re-obtain the optimal coupling position, with the aforementioned parameters recorded again for comparative analysis. Each position was tested ten times.

Building upon the static experiment, a dynamic experiment was subsequently conducted. By controlling the diffusely reflecting plate to move at a speed of 0.01 m/s within the water tank from near to far, the *P*_1_, *P*_2_ and *η* at corresponding positions were continuously recorded. This simulated a scenario in which LiDAR detects targets during underwater motion. The entire experimental process was repeated ten times.

### 4.2. Experimental Results

**Figure 16:**
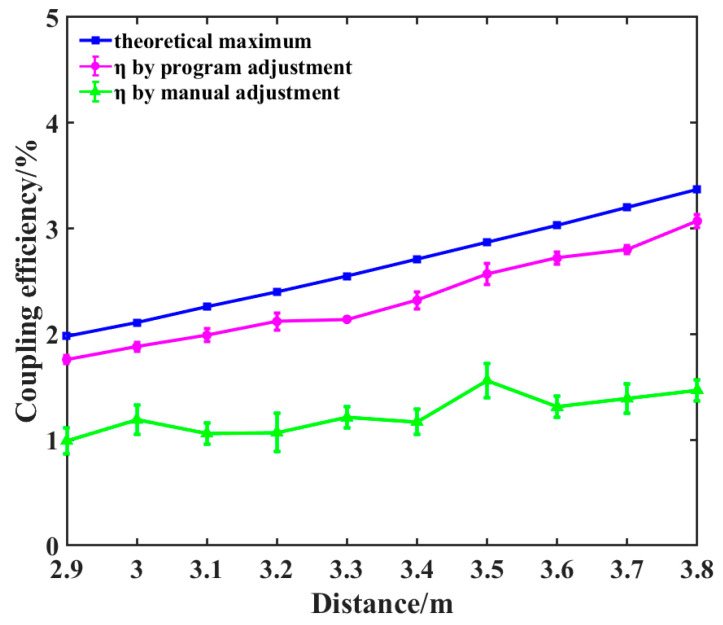
Comparison chart of static experimental results between program adjustment and manual adjustment (error bars indicate standard deviation).

**Figure 17:**
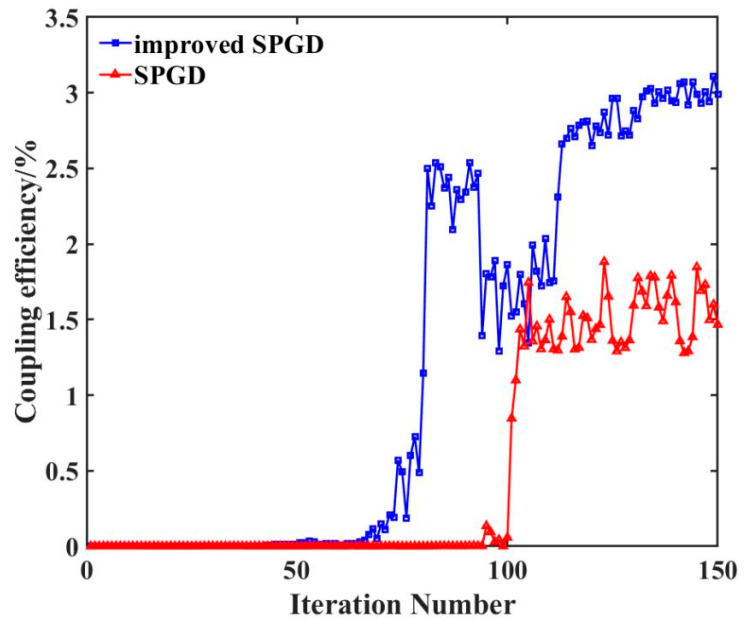
Performance comparison chart between the improved SPGD algorithm and the SPGD algorithm at a diffusely reflecting plate position of 3.8 m.

**Figure 18:**
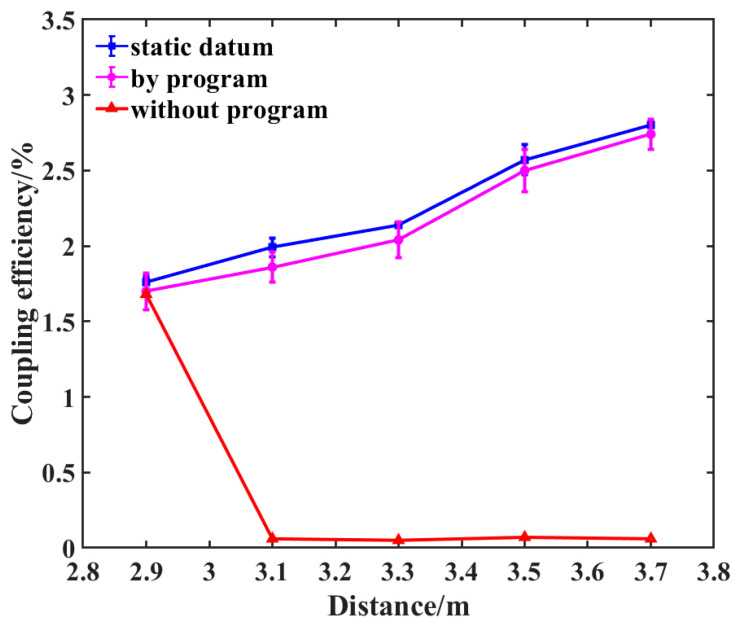
Comparison chart of dynamic experimental results with and without program adjustments (error bars indicate standard deviation).

Table 3 presents the spot size at the rear focal plane of the coupling lens, $\eta_{0}$, P_1_, and P_2_, where $\eta_{m}$ is the coupling efficiency of manual adjustment and $\eta_{p}$ is the coupling efficiency of program adjustment at different distances during static experiments. In the table, except for *η*_0_ and spot size, all other values are presented in the form of mean ± standard deviation. Figure 16 compares the optimal coupling efficiencies achieved through theoretical calculations, manual adjustment, and algorithmic adjustment. Figure 17 illustrates the performance difference between the conventional SPGD algorithm and the improved simulated annealing SPGD algorithm used in this paper when the target distance is set to 3.8 m. Figure 18 displays the variation in coupling efficiency during dynamic experiments with and without algorithmic adjustment.

In this experiment, after 2.9–3.8 m of propagation, the optical power incident on the fiber end face is about 3.04–3.31 μW (see Table 3), which is close to the typical power range (μW level to nW level) of weak echo signals in actual underwater LiDAR systems. If and when the echo signals are further weakened to nW levels and below, the system performance will be significantly affected by the low signal-to-noise ratio (SNR), which is mainly reflected in two aspects: (1) Impact on 4QD position detection accuracy: The detection sensitivity of 4QD is limited by its noise equivalent power. The 4QD selected in this paper has a responsivity of 0.28 A/W, a bandwidth of 100 kHz, and a response time of 35 μs. Under extremely low light conditions (<100 pW), the 4QD output signal may be swamped by noise, increasing the position detection error and, in turn, affecting the accuracy of rough alignment. (2) Impact on the power reading noise of the OPM and SPGD convergence: The power reading noise of the OPM directly affects the performance index of the SPGD algorithm, J. When the signal-to-noise ratio is reduced, the random fluctuations in J may mask small changes caused by the perturbation, leading to large errors in gradient estimation and affecting algorithm convergence.

As shown in Table 3 and Figure 16, when the distance of the diffusely reflecting plate varied between 2.9 m and 3.8 m, the average coupling efficiency $\eta_{m}$ achieved through manual adjustment was only 46.9% of the theoretical value $\eta_{0}$. Following program adjustment, however, the average coupling efficiency $\eta_{p}$ increased to 88.18% of the theoretical value $\eta_{0}$, representing an improvement of approximately 88% compared to the manual adjustment. The calculation formula for this improvement is [$(\eta_{p}-\eta_{m})/\eta_{m}$] × 100%. Moreover, the standard deviation of the coupling efficiency following program adjustment was markedly smaller than that observed after manual adjustment. The experimental results conclusively demonstrate that, under static conditions, the system can effectively compensate for alignment deviations, achieving an efficient, stable coupling of spatially scattered light.

As shown in Figure 17, when the diffusely reflecting plate is positioned 3.8 m away, the conventional SPGD algorithm tends to become trapped in local minima. In contrast, the improved simulated annealing SPGD algorithm effectively escapes local optima, ultimately achieving a coupling efficiency closer to the theoretical maximum. Furthermore, this algorithm exhibits an average convergence time of 0.3 s. This outcome demonstrates that incorporating the simulated annealing mechanism significantly enhances the algorithm’s global search capability while maintaining rapid convergence, thereby mitigating performance degradation caused by environmental disturbances or system noise.

As shown in Figure 18, when the diffusely reflecting plate moves slowly from near to far at a speed of 0.01 m/s, the coupling efficiency without program adjustment drops to nearly zero, indicating that manual or fixed alignment methods become entirely ineffective under dynamic conditions. However, after real-time adjustment using the program proposed herein, the coupling efficiency can be stably maintained at a level close to that observed in static experiments. This demonstrates that the system is not only suitable for static environments but can also achieve efficient fiber coupling through feedback control in dynamic environments where the target position continuously changes. However, in dynamic scenarios, the standard deviation of the average coupling efficiency after program adjustment is slightly greater than that observed in static scenarios. This indicates that both the system and the program still possess room for improvement.

## 5. Conclusions

This paper addresses the challenges of spatial scattered light coupling for single-mode fiber alignment in underwater lidar systems, which are characterized by difficulty and low efficiency. Relevant theoretical analyses and experimental research were conducted. By employing an extended light source imaging model, the coupling principle of spatial scattered light and its influencing factors were analyzed and simulated, revealing radial deviation as the most sensitive parameter. Building upon this foundation, an adaptive fiber coupling system incorporating reception, detection, and feedback control modules was designed and developed. An optimized control strategy combining rough and precise alignment was proposed: rough alignment utilizes 4QD for rapid beam pre-positioning, while precise alignment employs an improved simulated annealing SPGD algorithm to effectively avoid local optima, enhancing the convergence speed and stability. Experiments demonstrate that the system achieves an efficient coupling across varying target distances, with the coupling efficiency reaching 88.18% of the theoretical value—an 88% improvement over manual adjustment. It also maintains coupling efficiency under dynamic conditions, validating its applicability and reliability in complex underwater environments. This work presents an effective solution for coupling spatially scattered light in underwater LiDAR and related applications.

However, unlike real underwater environments, the experiments were conducted only under static tank and slow-moving target conditions and did not involve complex turbulent disturbances or fast-moving targets. In real ocean or lake environments, the inhomogeneous distribution of water temperature and salinity, as well as water movement, can lead to random fluctuations in the refractive index of the water body, causing intensity scintillation, undulation of the angle of arrival, and wave-front aberrations of the beam, and these effects can further affect the coupling efficiency. If the target moves too fast, the algorithm proposed in this paper may face the risk of convergence difficulty or even collapse, which in turn leads to a sharp decrease in the coupling efficiency. Future work will focus on the following aspects: (1) Turbulence simulation experiment: Build a tunable underwater turbulence simulation system (e.g., heating/stirring to generate controllable turbulence), systematically study the effect of different turbulence intensities on the coupling efficiency, and validate the robustness of this paper’s algorithm in turbulent environments. (2) Turbulence adaptive compensation: Explore the combination of turbulence statistical model predictive control strategies, using historical data to predict the deviation trend caused by turbulence, to achieve pre-compensation. (3) Algorithmic improvement: Use predictive SPGD algorithms or combine model predictive control strategies to further improve the algorithm’s convergence speed so that the algorithm can adapt to fast-moving target scenarios.

In addition, from laboratory prototypes to the deployment of actual underwater systems (e.g., AUVs, ROVs, and underwater LIDARs), there are still engineering issues to consider regarding system volume and power consumption. Currently, the system is about 700 mm × 300 mm × 300 mm, and the total power consumption is about 20–30 W. For medium- to large-scale underwater unmanned platforms, this size and power consumption index is still within an acceptable range. If further miniaturization is desired, it can be achieved by: (1) adopting an integrated controller with a compact size and low power consumption; (2) optimizing the optical path to simplify the system structure; and (3) adopting an embedded processing unit (e.g., FPGA or DSP) to replace the computer for the closed-loop control, which will reduce the power consumption and size of the system. Future research will also aim to engineer the integration and packaging of the system to improve environmental adaptability and conduct external lake or sea trials to verify the long-term stability and reliability of the system in actual underwater environments.

## Figures and Tables

**Table 1:** Main parameters of 532 nm single-mode fiber.

Parameters	Mode Field Radius	NA	Cladding Diameter	Coating Diameter
532 nmSMF	1.65 μm	0.12	125 ± 1.0 μm	245 ± 15 μm

**Table 2:** Main components and parameters of the module.

Module	Instrument Name	Core Parameters
Receiver module	Fast steering mirror	Caliber: 25.4 mm;
		dimensions: radial, angular;
		repeatability:
		radial < 0.105 μm;
		angular < 0.00004°;
		adjustment range: ±5°
	Combined lenses	Caliber: 25.4 mm;
		AR coating
		wavelength range: 350~700 nm;
		focal length: 150 mm
	Displacement stage	Standard cage: 25 mm;
		repeatability: 78 nm
Detection module	4-Quadrant Detector	Responsivity: 0.28 A/W;
		bandwidth: 100 kHz;
		wavelength range: 400~1100 nm;
		response time: 35 μs
	Optical Power Meter	Wavelength range: 400~1100 nm;
		power range: 500 pw~5 mw
Feedback control module	Controller 1	Communication method: USB;
		bits: 32 bit
	Controller 2	Bits: 32 bit;
		bandwidth: 168 MHz;
		communication method: USB

**Table 3:** Experimental results at different distances.

Distance/m	2.9	3.0	3.1	3.2	3.3	3.4	3.5	3.6	3.7	3.8
Spot size/μm	46.70	45.16	45.16	42.32	41.04	39.84	38.70	37.62	36.60	35.64
*η*_0_ /%	1.98	2.11	2.26	2.40	2.55	2.71	2.87	3.03	3.20	3.37
*P*_1_/μW	3.13 ± 0.03	3.20 ± 0.04	3.20 ± 0.05	3.18 ± 0.02	3.15 ± 0.05	3.23 ± 0.04	3.17 ± 0.03	3.16 ± 0.03	3.31 ± 0.07	3.04 ± 0.06
*P*_2_ from manual adjustment/μW	0.031 ± 0.007	0.038 ± 0.009	0.034 ± 0.012	0.034 ± 0.008	0.0385 ± 0.011	0.038 ± 0.009	0.0495 ± 0.010	0.0414 ± 0.008	0.046 ± 0.013	0.0447 ± 0.007
*η_m_*/%	0.99 ± 0.06	1.19 ± 0.07	1.06 ± 0.05	1.07 ± 0.09	1.21 ± 0.05	1.17 ± 0.06	1.56 ± 0.08	1.31 ± 0.05	1.39 ± 0.07	1.47 ± 0.05
*P*_2_ from program adjustment/μW	0.055 ± 0.003	0.06 ± 0.004	0.0637 ± 0.006	0.0674 ± 0.005	0.0674 ± 0.003	0.075 ± 0.006	0.0815 ± 0.005	0.086 ± 0.004	0.0927 ± 0.003	0.0933 ± 0.002
*η_p_* /%	1.76 ± 0.02	1.88 ± 0.02	1.99 ± 0.03	2.12 ± 0.04	2.14 ± 0.01	2.32 ± 0.04	2.57 ± 0.05	2.72 ± 0.03	2.80 ± 0.02	3.07 ± 0.03

## Data Availability

The data presented in this study are available on request from the corresponding author.
